# Harnessing artificial intelligence to enhance key surveillance and response measures for arbovirus disease outbreaks: the exemplar of Australia

**DOI:** 10.3389/fmicb.2023.1284838

**Published:** 2023-10-25

**Authors:** Andrew W. Taylor-Robinson

**Affiliations:** ^1^College of Health Sciences, VinUniversity, Hanoi, Vietnam; ^2^VinUniversity-University of Illinois Smart Health Center, VinUniversity, Hanoi, Vietnam; ^3^Center for Global Health, Perelman School of Medicine, University of Pennsylvania, Philadelphia, PA, United States; ^4^College of Health and Human Sciences, Charles Darwin University, Casuarina, NT, Australia

**Keywords:** Research topic: Transmission and infection of arboviruses, surveillance, response, outbreak, public health, artificial intelligence, Australia

## 1. Contributions to the field statement

Arboviruses present a significant public health risk to the Australian population. Both the many indigenous arboviruses and imported cases of major global pathogens contribute to this burden. Effective surveillance measures, which involve monitoring for mosquitoes responsible for transmission, the signs and symptoms of disease in humans, and a range of environmental and climactic factors, are essential to detect and respond early to local outbreaks. This is particularly crucial in regional Australia, a vast area that is underserved but is now becoming a focal point for economic and social development. As this transformation progresses, there will be increased human interaction with native reservoir animal hosts and vector mosquitoes, creating a potential scenario for a higher prevalence of neglected indigenous arbovirus infections. Additionally, the impact of climate change in the tropical north of the country is predicted to lead to a population boom of arbovirus-transmitting mosquitoes, further exacerbating the situation. Hence, it is imperative to maintain diligent attention to vector monitoring and control efforts. Integrating artificial intelligence to rapidly process large volumes of data should enhance surveillance by improving data analysis, prediction, and decision-making. More accurate and quicker detection of arboviral disease outbreaks will enable proactive and effective public health responses.

## 2. Introduction: the public health problem

Viruses that are transmitted between vertebrate hosts by biting, blood-feeding arthropods (primarily mosquitoes and ticks) are called *ar*thropod-*bo*rne viruses or, for short, arboviruses. The transmission of arboviruses to humans poses a significant and accelerating global public health risk (Madewell, 2020). It is estimated that 3.9 billion people, approaching half of the world's population is at risk (World Health Organization, 2022), leading to hundreds of millions of symptomatic infections, a disease burden of tens of thousands of deaths and up to 5 million disability-adjusted life years lost annually (Labeaud et al., 2011). Notable examples of pathogens include dengue (DENV), chikungunya, yellow fever, Japanese encephalitis (JEV), West Nile, Zika and Mayaro viruses. For several of these, humans serve as the primary reservoir host, with many causing pandemics over the last few decades (Mayer et al., 2017). Mild infection is typically associated with influenza-like symptoms such as fever, headache, muscle or joint pain, and/or a skin rash. Less commonly, severe infection is characterised by rapid onset of haemorrhagic fever (with internal bleeding) or life-threatening shock syndrome (with circulatory collapse). Signs and symptoms of encephalitis include confusion, tremors, seizures, paralysis, and loss of consciousness (Labeaud et al., 2011; Mayer et al., 2017).

The distribution of an arbovirus is restricted by the territory of its mosquito vector(s) of transmission, which tends to be limited to tropical and subtropical zones. Yet, due to the effects of climate change (involving rainfall patterns) the geographical range of common vectors may be predicted to expand in future (Madewell, 2020). Thus, locations that at present are currently not affected should not be complacent that they will always remain free of arboviruses. The dramatic emergence and re-emergence of arboviral diseases has been greatly exacerbated by a combination of global meteorological, demographic, and societal changes, principally increasing rates and levels of climate change, urbanisation, globalisation, and international mobility (Bellone et al., 2023). These environmental and anthropogenic factors have facilitated viral etiological agents to break out of their natural ecological zones to become established in novel geographical sites where susceptible arthropod vectors and human hosts provide conditions supportive to their causing epidemics (Madewell, 2020).

## 3. The usual suspects

DENV is an arbovirus of global concern but for which local outbreaks in Australia are restricted to Queensland, where the vector mosquito *Aedes aegypti* is established (Beebe et al., 2009). Community acquired infections have been reported only from urban areas in the northeast of the state, where the vector is most abundant. However, historical data show that much of Australia has previously sustained both the virus and the vector mosquito (Russell et al., 2009). Factors such as increased DENV activity in neighbouring countries like Indonesia and Papua New Guinea, plus the growing human population of northern Australia contribute to the risk of DENV transmission (Gyawali et al., 2016a). Climate change projections also suggest potential rises in dengue incidence and distribution associated with increasing temperatures (Williams et al., 2014). This also applies to JEV, the recent and rapid emergence of which in several states is a cause for concern (Williams et al., 2022).

Imported cases of DENV and other arboviruses, including JEV, also pose a risk to public health in Australia. With increased global travel and trade, there is a potential for the introduction of arboviruses through infected travellers or imported vectors (Mackenzie and Williams, 2009). The spread of arboviruses to regions without established vectors, such as *Ae. aegypti* and *Ae. albopictus*, can occur through international air and sea ports (Gyawali et al., 2016a). Therefore, surveillance and control measures at ports of entry are crucial to prevent the importation and establishment of arboviruses in Australia. The most recent national report, for 2016, shows 2,227 notifications of DENV, of which 31 were locally acquired and the remainder travel-related, mostly tourists visiting Bali (Australian Government Department of Health, 2021).

## 4. The less usual suspects

The threat presented by emerging indigenous arboviruses in Australia is arguably undervalued (Gyawali et al., 2016b). More than 75 arboviruses have been identified that are unique to the continent. While several are recognised to cause disease in humans, information on the potential human pathogenicity of most of these indigenous viruses is negligible (Gyawali et al., 2017a). Ross River (RRV) and Barmah Forest (BFV) viruses trigger an often debilitating and sometimes chronic type of arthritis that affects several joints at once. Murray Valley encephalitis (MVEV) and West Nile Kunjin strain (KUNV) viruses cause inflammation of the brain.

One of the key arboviruses of concern is RRV, which is endemic and enzootic in the country and Papua New Guinea (Kuleshov et al., 2022). The major vector in inland areas is the freshwater-breeding *Culex annulirostris*, whereas *Ochlerotatus vigilax* and *O. camptorhynchus* transmit in brackish coastal waters. RRV infection in humans can cause peripheral polyarthralgia or arthritis, with disease notifications averaging 5,000 per year in Australia since the start of this century. Yet, there is considerable annual fluctuation of confirmed case reports; for instance, 9,555 notifications in 2015 but 3,677 in the following year (Australian Government Department of Health, 2021).

As with RRV, human infections with BFV have been reported from all states and territories in Australia. Moreover, serological surveys indicate that this is a widespread phenomenon. Clinical manifestations often include fever, rash, chronic fatigue and polyarthritis. BFV is transmitted primarily by *Cx. annulirostris* and *Aedes funereus* in inland and in coastal regions, respectively. The reported incidence is usually close to 1,000 cases per annum since routine testing by immunoassay antibody detection became widely available (Gyawali and Taylor-Robinson, 2017).

MVEV is endemic in northern Australia, with sporadic outbreaks occurring (Broom et al., 2003). The virus is transmitted primarily by *Cx. annulirostris* mosquitoes, and its activity is influenced by rainfall and flooding. Other emerging arboviruses, such as KUNV, have been detected in this and other ornithophilic mosquitoes and pose a potential public health threat (Broom et al., 2003). The presence of competent vectors and the potential for virus introduction through travel and trade increase the risk of emerging indigenous arboviruses in Australia (Mackenzie and Williams, 2009).

Other Australian arboviruses, such as Alfuy, Edge Hill, Gan Gan, Kokobera, Sindbis and Stratford, are also associated with human disease (Gyawali et al., 2019). However, they appear to cause predominantly mild symptoms and a major outbreak has not yet been reported. While the epidemiology of these neglected viruses is poorly understood, they are likely maintained in zoonotic cycles rather than by human-to-human transmission. Hence, they are harboured by apathogenic, persistent infections in native Australian reservoir mammals (such as kangaroos and wallabies) and birds (including herons and egrets) (e.g., Gyawali et al., 2020), with occasional spillover into humans.

## 5. Need for improved early detection

For many years it was speculated that infection with arboviruses may be a cause of febrile illness in Australia, as elsewhere in the world. This was confirmed with the discovery of the now frequently diagnosed RRV in 1959 and BFV in 1974. Yet, even after identification of these viruses it took almost 15 years for routine laboratory tests (involving detection of virus-specific immunoglobulin (Ig) M and IgG) to diagnose infection to become available (Gyawali et al., 2017a). While paired serology of RRV and BBV is considered clinical best practise, it requires careful interpretation considering the high rates of false positive and negative results, plus the long-term persistence of IgM in some individuals. Incorrect interpretation risks misdiagnosis and therefore inappropriate patient treatment (Gyawali et al., 2017b).

Compounding this problem of inaccurate viral infection case reporting is the fact that more than half of so-called undifferentiated fevers (those with non-specific symptoms) in Australia still go undiagnosed (Gyawali et al., 2017a). In many instances this is because the treating physician may consider the cost of testing is not justified or the causative agent is novel, not known to cause human disease or no routine diagnostic test is available. In such cases, an association could be assumed but not proved between arboviruses and feverish illness. Hence, establishing a robust surveillance system would enable the early warning of an infection outbreak. Developing accurate diagnostic tools would aid early diagnosis and correct treatment of febrile primary care patients.

Unforeseen climatic and environmental variations, such as the increased incidence of cyclones, heavy rainfall, and resultant intensified flooding associated with outbreaks of RRV (Tall et al., 2014) and MVEV (Selvey et al., 2014), have been occurring of late with disconcerting regularity, potentially effectuating an ecological change for Australian arboviruses (Young, 2018). The projected future climatic suitability of Northern Australia for competent vector mosquito species needs to be evaluated. In this context, improved epidemiological surveillance of prevailing environmental conditions, mosquito vector species and reservoir host animals, should be considered a public health priority.

## 6. A proposed solution

In order to prepare effectively for the emergence of an arbovirus outbreak of public health concern, both globally (Weaver and Reisen, 2010), and in particular in regional Australia (Gyawali and Taylor-Robinson, 2017), key surveillance measures are essential. These include the following five actions:

1. *Vector surveillance*: monitoring and mapping the distribution and abundance of mosquito vectors is crucial. This involves regular trapping and identification of vector species, as well as testing them for the presence of arboviruses. Vector surveillance helps identify areas at risk and informs targeted control measures.
2. *Environmental surveillance*: monitoring environmental factors, such as temperature, rainfall, and humidity, can provide insights into vector breeding and arbovirus transmission dynamics. This information helps predict and anticipate outbreaks, enabling timely interventions.
3. *Animal surveillance*: monitoring arboviral infections in animal populations, particularly in sentinel species, can serve as an early warning system for human outbreaks. Animals, such as marsupials and water birds, can act as reservoir hosts or environmental indicators of arbovirus activity.
4. *Disease surveillance*: active surveillance for human cases of arboviral infections is vital. This involves monitoring and reporting suspected cases, conducting microbiology laboratory testing for confirmation, and analysing epidemiological data to identify trends and patterns. Early detection and reporting of cases allow for prompt public health responses.
5. *Syndromic surveillance*: implementing surveillance systems that monitor specific clinical symptoms or syndromes associated with arboviral infections can provide early indications of outbreaks. Health indicators that are discernible before confirmed diagnosis include monitoring febrile illnesses, neurological symptoms, and other relevant clinical presentations.

## 7. A novel approach

Artificial intelligence (AI) can play a prominent role in enhancing arbovirus surveillance at scales ranging from local to global. AI algorithms can analyze large volumes of data, including environmental, epidemiological, and entomological data. Integrating human, pathogen, vector, and climatic variables from various existing surveillance sources into a unified system can enhance pattern recognition and generate probabilistic risk models for outbreak spread and severity (Pley et al., 2021). This allows epidemiologists to detect patterns, predict outbreaks, and inform targeted interventions more accurately using such high-throughput techniques as metatranscriptomic sequencing (Batovska et al., 2022). Moreover, AI can automate data processing, improve data integration, assist in modelling, and provide real-time monitoring and analysis of multiple variables. This enables public health authorities to identify areas at high risk, to allocate resources more efficiently and thereby to make more proactive and effective responses (Batovska et al., 2019; Pley et al., 2021).

In the context of arbovirus surveillance, AI can assist in the identification and tracking of mosquito vectors, processes that are crucial for understanding the transmission dynamics of arboviruses. By analysing data on mosquito populations, AI algorithms can identify trends and patterns that may indicate increased virus activity or the novel emergence of a virus in a location (Ramírez et al., 2018). This information can then be used to guide control and preventive measures. For example, AI can analyze satellite imagery and climate data, mosquito surveillance data, and human case data to reveal vector habitats, identify high-risk areas for disease outbreaks, and predict disease transmission dynamics (Kurucz et al., 2022). AI algorithms can also analyze social media and internet search data for disease outbreaks and public concerns to help to develop early warning systems and decision support tools (Batovska et al., 2022). Additionally, AI can assist in data integration and modelling to improve disease forecasting and inform targeted interventions by public health authorities.

Research conducted in Kenya has demonstrated the effectiveness of mosquito-based arbovirus surveillance in diverse ecological zones (Ochieng et al., 2013). Similarly, in Burkina Faso, AI has been employed to enhance surveillance during dengue outbreaks, leading to improved understanding of the burden of arboviral diseases (Sanou et al., 2018). These experiences highlight the potential of AI in strengthening surveillance and response measures for arbovirus disease outbreaks globally. Therefore, the use of AI in arbovirus surveillance is not limited to Australia. Yet, this affluent developed nation is particularly suited to integrating AI into outbreak preparedness procedures (van den Hurk et al., 2012). It has the infrastructure and resources to leverage AI better than most countries, while also having plenty to gain by mitigating a neglected public health threat, especially in rural and regional locations that are relatively underserved.

## 8. Conclusion

Arboviruses pose a significant public health threat to the population of Australia. There is a risk of emerging indigenous arboviruses, while imported cases also contribute to the burden. Surveillance measures, including monitoring vectors, diseases, and environmental factors, are crucial for early detection and response to outbreaks. This is particularly impactful in regional Australia, a historically underinvested region that is set to become a focus of economic and social development. This will increasingly bring humans into close contact with native reservoir hosts and vector mosquitoes. Such a convergence of factors could trigger an increased prevalence of infection with neglected indigenous arboviruses. Moreover, the escalating rate and effects of climate change that are observed in the tropical north of the country will likely drive a population boom of arbovirus-transmitting mosquitoes. As a commensurate response, continuing assiduous attention to vector monitoring and control is required. It is anticipated that the integration of artificial intelligence to process large volumes of data rapidly will enhance surveillance efforts by improving data analysis, prediction, and decision-making, ultimately leading to improved accuracy in detecting arboviral disease outbreaks and enabling more proactive and effective public health responses. The lessons learned from this Australian experience can help to better prepare government agencies in other nations to adopt AI technology in their enhanced surveillance efforts. In particular, this applies to low- and lower-middle income countries in tropical and subtropical zones where the rising incidence of arboviral diseases is a major public health concern.

## Author contributions

AWT-R: Conceptualisation, Formal analysis, Investigation, Writing—original draft, Writing—review and editing.
